# Medication non-adherence and its associated factors among kidney transplant patients in a large teaching hospital in Ethiopia

**DOI:** 10.1186/s12882-024-03620-z

**Published:** 2024-06-01

**Authors:** Meskerem Nimani Derejie, Erimas Nimane Dereje, Dirijit Mamo Alemu, Yemane Gebremedhin Tesfay, Fufa Hunduma, Negash Miniwye Temie

**Affiliations:** 1 General Public Health, St.Pauìs Hospital Millennium Medical College, Addis Ababa, Ethiopia; 2 Addis Ababa Burn Emergency and Trauma Hospital, Addis Ababa, Ethiopia; 3 Emergency Medicine and Critical Care, St.Pauìs Hospital Millennium Medical College, Addis Ababa, Ethiopia; 4 Feild Epidemiology, St. Pauìs Hospital Millennium Medical College, Addis Ababa, Ethiopia; 5 Oshakati Hospital, Oshakati, Namibia

**Keywords:** Medication non-adherence, Associated factors, Post-kidney transplant, A large teaching hospital, Ethiopia

## Abstract

**Background:**

This study examines medication adherence among kidney transplant patients at St. Paul’s Hospital Millennium Medical College (SPHMMC) in Addis Ababa, Ethiopia, focusing on the level of adherence and associated factors to immunosuppressant medicines.

**Methods and materials:**

A cross-sectional study was conducted on 270 patients from October 2021 to January 2022 using a structured questionnaire analyzed with SPSS version 26. The prevalence of medication adherence was computed, and a binary logistic regression was fitted to estimate the association. Medication adherence level measurement in post-kidney transplant patients was assessed using the Simplified Medication Adherence Questionnaire (SMAQ) and Basel Adherence Assessment Scale in Immunosuppressants (BAASIS). A 95% confidence interval and *p*-value < 0.05 were used for statistical significance.

**Results:**

The study found that 71.5% of kidney transplant patients were male, with a median age of 37 and a mean duration of 3.55 years. Medication adherence in post-kidney transplant patients was 81.9%. Post-transplant duration above 5 years and missing follow-up visits more than two times was associated with a 92.6% and 91.2% in medication non-adherence rate respectively. Additionally, forgetfulness was associated with a 90.6%, non-adherence level compared to drug unavailability and financial reasons.

**Conclusion and recommendation:**

The study indicates that our patients exhibit higher medication adherence than WHO-measured levels, suggesting the need for healthcare providers to strengthen their intervention, especially for those above 5 years post-kidney transplant. The reason for increased adherence could be explained by the health education program about the medication name, dosing, frequency of ingestion and adverse effects of the drug, and effects of non-adherence.

## Background

Medication burden, very low income and being a farmer were associated with medication non-adherence among CKD ( Chronic Kidney Disease) and ESRD( End Stage Renal Disease) in Ethiopia [1].

Kidney transplantation (KT) is the treatment of choice for end-stage renal disease (ESRD) due to its survival, quality of life, and cost-effectiveness [2].

Although renal transplant services require structural organization, various expertise, and advanced laboratory services, Ethiopia is one of the few African countries managed to have the service since 2015 [3, 4]. I t is located in one of the teaching hospital in Addis Ababa, St. Paul’s Hospital Millennium Medical College. It has transplanted 148 patient, which has follow up at the transplant unit in addition to patients that had KT abroad [4]. Health education program is given about the transplantation process, medication used and laboratory intervention [4, 11]. 

The Global Observatory on Donation and Transplantation, in collaboration with WHO, states that organ transplantation is the only treatment for end-stage failure of solid organs, requiring lifelong immunosuppression medication to prevent rejection. The first-year graft rejection rate varies from 7 to 36%. The increasing prevalence of CKD and ESRD, shortage of donors, limited transplantation services, and health-related costs make preventing adverse outcomes crucial [3–5].

To prevent graft failure and life-threatening, costly complications, transplant recipients must closely adhere to their medication regimens, which require constant monitoring of therapeutic levels [6].

However, adherence to immunosuppression medications is suboptimal in high-income countries (HICs) and lower in low- and middle-income countries (LMICs). Non-adherence to medication regimens increases the risk of rejection, kidney loss, and costly treatments, which poses challenges in resource-limited settings [8].

HIC countries offer advanced kidney transplantation services, easy medication access, and strong health insurance. However, LMIC countries face limited KT services due to shortages of nephrologists, transplant centers, and high immunosuppressive medication costs, making patient management challenging [3, 9].

A consistent supply of immunosuppressive medication is crucial for transplantation survival, but availability and affordability pose significant barriers to successful transplant program development [10].

This research aims to determine patient adherence status among KT patients at St.Paul’s Hospital Millennium College, and its associated factor.

## Methods and materials

### Study area and period

The study was conducted at the St. Paul`s Hospital Millennium Medical College (SPHMMC) National Kidney Transplant Center in Addis Ababa, Ethiopia. Established in 2013, the unit is the first kidney transplant service in the country. Since 2015, it has performed around 148 transplants for chronic kidney disease (CKD) and end-stage renal disease (ESRD) patients. The center provides clinical follow-up services for more than 400 indigenous KT patients and those transplanted abroad. The study was conducted from October 12th, 2021, to January 4th, 2022.

### Study design

A cross-sectional study was conducted on patients who underwent kidney transplantation at the SPHMMC renal transplant unit and abroad and who underwent clinical follow-up at the unit during the study period.

### Source and study population

The study focuses on CKD and ESRD patients who have undergone kidney transplants in the SPHMMC renal transplant unit or abroad. There are 148 patients with KT service and 252 post-KT patients who received service abroad and have clinical follow-ups at the renal transplant clinic. Despite 400 post-KT patients starting clinical follow-up at the clinic, some discontinued it. The study population includes all post-KT patients who meet the inclusion criteria during the study period.

### Sample size determination and sampling procedures

A purposive sampling method was used to study the small, well-defined, and unique source population. All eligible patients who had their clinical follow-up at the SPHMMC renal transplant unit during the study period were included. The study excluded patients who were lost from follow-up, deceased, or transplant rejected and were on dialysis. Data was collected during clinical follow-up appointments, which varied depending on the time of transplant and clinical condition. 270 patients were accessed and responded to, with a data collection period of two and a half months from October 12, 2021, to January 4, 2022.

### Eligibility criteria

#### Inclusion criteria

All post-kidney transplant patients having follow-up service at SPHMMC renal transplant unit and are willing to participate in the study.

Patient recruited as they came for follow-up visit, and with that all the eligible patients will be accessed in the study period.

### Exclusion criteria

The study excluded patients who are critically ill, unwilling to participate, inaccessible, under 18 years old, or with failed transplants or transplant rejections, and those on dialysis.

#### Study variables

**Dependent variables**.

- Medication adherence in post kidney transplant Patients.

**Independent variables (five WHO dimensions)**.

A. Social and economic:

- Cost (treatment cost, transportation cost): occupation or income source.
- Sociodemographic variable: age, sex, educational background and marital status.

B. Health system related: patient to health care provider interaction, and health care provider follow up and multiple health care provider.
C. Condition related: these are characteristics of the disease, disease condition/severity and patient related health literacy:
D. Patient perceived importance of medication,: cognitive functioning, self-care motivation and social support.
E. Therapy related: Multiple medication, Complexity of therapy and Adverse drug reactions, and duration of therapy.

### Data collection tools and procedures

#### Data collection tools

A structured questionnaire was used to measure medication adherence, adopting and modifying the simplified medication adherence questionnaire (SMAQ) six items. The Basel assessment of adherence to immunosuppressive medication scale (BAASIS) questionnaire was used for specific immunosuppressive medications. The questionnaire was translated into Amharic for easy comprehension by participants and trained data collectors.

### Data collection procedures

The questionnaire was tested with 10 participants on October 8th, 2021, and modified after. Data collection was conducted from October 12th to January 4th, 2022. Confidentiality was maintained through anonymity and secure storage.

### Data quality management

Data quality was ensured by selecting general practitioners as data collectors, training them on the procedure, and ensuring consistency. The principal investigator managed data cleaning, encoding, and analysis using Epi Info 7 software and SPSS version 26.

### Data processing and analysis

The collected data was processed and cleaned until January 9, 2022.

The Simplified Medication Adherence Questionnaire (SMAQ) was used to score the prevalence of medication adherence.

The magnitude of adherence was calculated using the SMAQ scale scoring for each patient. The BAASIS questionnaire was used to measure non-adherence to immunosuppressant medications over the past four weeks.

A chi-square test was run to compare the underlying characteristics of patients based on adherence status.

Binary logistic regression analysis was used to analyze different factors for medication adherence. Bivariate analysis was used to measure the association between the dependent variable and each independent variable. Independent variables with a *P* < 0.25 were considered for multivariate analysis. Multicollinearity was tested with a linear regression model for the independent variables.

The goodness of model fit test was done by the Hosmer and Lemeshow tests. The multicollinearity of the independent variables was tested by a linear regression model.

The findings and conclusions were discussed, with possible interventional tools and recommendations.

### Operational definitions

#### Immunosuppressive medication

medications that are prescribed for the purpose of preventing graft rejection in the kidney transplantation.

#### Adherence

the extent to which a person’s behavior in taking medication, following a diet, and/or executing lifestyle changes, corresponds with agreed recommendations from a health care provider.

#### Adherent/ complaint

if he/she responds to all of the questions with adherent answer and in terms of quantification, if the patient has responded with adherent answer in the forgetfulness, routine, adverse effect and quantifying assessment questions by using a SMAQ questionnaire score [23]. 

#### Non-adherent/non-compliant

if he/she responds to any of the questions with a non-adherence answer, and in terms of quantification, if the patient has lost more than two doses during the last week or has not taken medication during more than two complete days during the last three months by SMAQ questionnaire score [23]. 

### Ethical considerations and consent to participate

Ethical clearance to conduct the study and official letter was obtained from the institutional review board of SPHMMC. After explanation about the whole purpose of the study, written informed consent was obtained from study participants.

All of the study participants were informed about the purpose of the study, about their right to participate or to terminate at any time if they want and also respondents were ensured about the confidentiality of information obtained.

The authors declare that all the methods included in the study are in accordance with the declaration of Helsinki.

## Results

### Socio-demographic characteristics

From 296 patients who were still following at the renal transplant center, information was collected from 270 patients making the response rate 91.5%. The median age of the patients was 37 (IQR, 28–45) years. More than two-thirds of the patients 193 (71.5%) were males. Most 137 (50.7%) of the patients were educated having a first degree and above. Most, 178 (65.9%), of the patients were married. The summary of the sociodemographic results is in Table 1.

**Table 1:** Sociodemographic characteristics of post kidney transplant patents in (*N* = 270) SPHMMC

Variable		Frequency (*n*)	Percentage (%)
Age group	≤ 30	90	33.3
	31–45	114	42.2
	> 45	65	24.2
Sex	Male	193	71.5
	Female	77	28.5
Educational background	Primary school	21	7.8
	Secondary school	89	33.0
	Certificate and diploma	23	8.5
	First degree and above	137	50.7
Marital status	Single	90	33.3
	Married	178	65.9
	Widowed	2	0.7
Religion	Orthodox Christian	183	67.8
	Muslim	59	21.9
	Protestant	27	10.0
	Other	1	0.4
Occupation	Governmental employee	66	24.4
	Non-governmental employee	30	11.1
	Private business	118	43.7
	No job	56	20.7

N-number of patients, SPHMMC- Saint Paul`s Hospital Millennium Medical College.

### Patient related characteristics of post kidney transplant patients

The median duration of illness started was 5.6 (IQR = 3.4–7.2) years; and the duration after transplant was 3.1 (IQR = 1.2-5.0) years. However, the respondent’s minimum duration after transplant was 1 month and the maximum duration was 18 years (Range = 17.92 years).

### Treatment related characteristics of post kidney transplant patients

Most patients grade the renal transplant service and interactions with their health care provider as very good, with 94.8% and 95.9%, respectively. The majority 258 (95.6%) of the patients were cared by the same doctor in each of their visits.

Almost all 269 (99.6%) of the patients had detailed explanations given by the health care provider about their medication and 268(98.5%) of them responded that they had the knowledge too. The average number of drugs taken by the patients was 5.21 (SD ± 1.81) drugs with minimum of 2 drugs and a maximum of 11 drugs and almost all 268 (99.3%) were taken twice per day. The most frequent answer given a reason for non-adherence was forgetfulness 164(60.7%), followed by drug unavailability 60 (22.2%). ( The detailed summary of the patient and treatment related factor analysis result is in Table 2).

**Table 2:** Disease, patient, treatment and health setup related characteristics of post kidney transplant patients (*N* = 270) in SPHMMC

Variables		Mean(± SD) in years	Median (IQR) in years
Duration after transplant			3.1(1.2-5.0)
Number of drugs		5.2(1.8)	
		Frequency (n)	Percentage (%)
Duration after transplant	≤ one-year	64	23.7
	> 1–5 years	152	56.3
	> 5 years	54	20
Service given	Good	5	1.9
	Very good	256	94.8
	Excellent	9	3.3
Interaction between patient and HCP	Good	2	0.7
	Very good	259	95.9
	Excellent	9	3.3
Encounter the same HCP always	Yes	258	95.6
	No	12	4.4
Having different HCP in each visit affects medication adherence	Yes	28	10.4
	No	242	89.2
Does HCP explain the medication in details	Yes	269	99.6
	No	1	0.4
Physical limitation	Yes	8	3.0
	No	262	97.0
Time to seek care During illness	Within a day	206	76.3
	2–5 days	64	23.7
Have Social support	Yes	267	98.9
	No	3	1.1
Follow up importance	Yes	268	99.6
	No	1	0.4
Did you ever Miss follow up?	Never	146	54.1
	one time	47	17.4
	two times	41	15.2
	> two times	36	13.3
Medication prescribed importance	Importance	159	58.9
	Very importance	111	41.1
Have Knowledge about your medication	Yes	266	98.5
	No	4	1.5
Reason for non-adherence	Drug Unavailability	60	22.2
	Drug adverse effects	21	7.8
	Financial shortage	17	6.3
	Forgetfulness	164	60.7

HCP- Health Care Professionals, IQR- Inter Quartile Range, N-number of patients, SD- Standard Deviation, SPHMMC- Saint Paul`s Hospital Millennium Medical College.

### Prevalence of medication adherence among post kidney transplant patients

Among 270 participants, 221(81.9%) were found to be adherent (with CI = 0.767,0.863) by using SMAQ adherence measurement scale with six questionnaire tools, as summarized in the table for each specific answer frequency. Based on BAASIS measurement tool [28–30], which is specifically designed for immunosuppressant medication adherence measurement by the University of Basel, about 208(77.0%) were adherent to their immunosuppressant medication (with CI = 0.816, 919). All patients had a response of that immunosuppressant medications are essential or important. Most 164(60.7%) of the patients think forgetfulness is the reason for medication non-adherence. The specific SMAQ and BAASIS measurement is summarized in Table 3 below.

**Table 3:** Medication adherence level measurement in post kidney transplant patients using Simplified Medication Adherence Questionnaire (SMAQ®) and Basel Adherence Assessment Scale in Immunosuppressant (BAASIS®)

Variables			*N* (%)
SMAQ®	Do you always take your medication at the appropriate time?	Yes	221(81.9%)
		No	49(18.1%)
	When you feel worse/bad, have you ever discontinued taking your medication?	Yes	11(4.1%)
		No	259(95.9%)
	Do you ever forget to take your medication?	Yes	179(66.3%)
		No	91(33.7%)
	Did you not take any of your medicine over the past weekend?	Yes	37(13.7%)
		No	233(86.3%)
	Thinking about the last week. How MANY TIMES did you fail to take your prescribed dose?	Never	231(85.6%)
		1–2 times	32(11.9%)
		> 2 times	7(2.6%)
	Over the past 3 months or since your last visit (appointment), how many days have you not taken any medicine at all?	Never	96(35.6%)
		1–2 times	127(47%)
		> 2 times	47(17.4%)
Adherence: SMAQ score		221(81.9%)	
BAASIS®	Intake non-adherence	Never	246(91.1%)
		1/month	22(8.1%)
		1/2 weeks	2(0.7%)
	Drug Discontinuation	Never	264(97.8%)
		1/month	6(2.2%)
	Time non-adherence	Never	224(83.0%)
		1/month	35(12.9%)
		1/2 weeks	6(2.6%)
		Every week	3(1.1%)
		> 1/week	2(0.7%)
	Dose alteration	Never	270(100%)
		1/month	0(0)
Adherence: BAASIS score		208(77.0%)	

BAASIS-Basel Assessment of Adherence to Immunosuppressive Scale, N-Number of patients, SMAQ-Simplified Medication Adherence Questionnaire, SPHMMC- Saint Paul`s Hospital Millennium Medical College.

### The associated factors that affect medication adherence in post kidney transplant patients

The medication adherence was found to be 81.9% with a 95% CI (0.767, 0.863), a binary logistic regression model with bi-variate analysis was used to identify the association between adherences with the candidate variables. With a level of significance below 25% for crude odds ratio analysis of age, marital status, duration after transplant, missed follow-up, drug unavailability, drug adverse effects and forgetfulness were associated with medication adherence. The age group above 45 years had a lower medication adherence level by 67.4% (with COR 0.326, a *p*-value of 0.12) as compared to age 37 years and below. Based on the duration of years after transplant, as the duration increases, the level of adherence decreases by 13.3% with a statistical significance (COR 0.867, *p*-values 0.006). The duration after transplant above five years had 93.5% less adherence level as compared to one-year and below duration after transplant years (COR 0.065, *p*-value < 0.0001). Missed follow-up was also found to be a significant factor from bi-variate analysis; those who missed follow-ups more than two times were associated with a 90.8% decreased adherence level as compared to those who never missed follow up visits (COR 0.092, *p*-value < 0.0001). There were different reasons responded by the patients for medication non-adherenc: forgetfulness was associated with decreased level of adherence by 90.7% as opposed to drug unavailability response given by the patients (COR 0.103, *p*-value 0.002).

There was no collinearity or dependency detected among the independent variables from one to another, with tolerance < 0.2 and VIIF < 510. Then a multivariable binary logistic regression was done to refine the associated factors from possible confounders among the predictor variables with a *p*-value < 0.25 level of significance in the bi-variate analysis.

By using a backward logistic regression model at a 5% level of significance, missing follow-up, duration after transplant, and forgetfulness were found to have a significant association with medication adherence. Accordingly, after adjusting for other covariates, those patients who had missed follow-up visits once had the odds of a 69.9% decreased level of medication adherence as compared to those who never missed follow up (AOR 0.301, *p*-value 0.032). And those patients who had missed follow-up visits two times had the odds of 82.4% decreased level of medication adherence as compared to who never missed follow up (AOR = 0.176, 95%, *p*-value 0.001); and similarly, those patients who had missed follow up visits more than two times had the odds of 91.2% decreased level of medication adherence as compared to who never missed follow up (AOR 0.088, *p*-value < 0.0001).

Concomitantly, those who had duration after transplant above five years had 92.6% less adherence as compared to those with duration after transplant one year and below (AOR 0.074, *p*-value 0.031). Similarly, forgetfulness was associated with an odds of 90.6% decreased medication adherence level as compared to other reasons provided for non-adherence (AOR 0.104, *p*-value 0.006). But the other factors age, grouped age, after transplant years and marital condition which had an association with the crude odds ratio, didn’t show a significant association with medication adherence when analyzed by multivariate analysis. (The detailed summary of the associated factor multiple regression result is in Table 4).

**Table 4:** Bivariate and multivariable analysis of factors associated with medication adherence among post kidney transplant patients (*N* = 270) in SPHMMC

Variables		Adherence		COR (95%, CI)	AOR (95%, CI)	*P*-value
		Adherent *N*(%)	Non-adherent *N*(%)	Bivariate	Multivariable	
Age				0.967(0.95–0.997)	1.05(0.964–1.14)	0.28
Age group	≤ 30 years	80(88.9)	10(11.1)	1(0)	1(0)	
	31–45 years	94(82.5)	20(17.5)	0.588(0.26–1.33)	0.442(0.109–1.78)	0.251
	> 45 years	47(72.3)	18(27.7)	0.333(0.14–0.77)	0.19(0.016–2.22)	0.185
Marital status	Unmarried	82(89.1)	10(10.9)	1(0)	1(0)	
	Married	139(78.1)	39(21.9)	0.435(0.21–0.92)	0.682(0.22–2.07)	0.499
Duration after transplant				0.867(0.78–0.96)	1.17(0.918-1.50)	0.201
Duration after transplant category	< 1 year	62(96.9)	2(3.1%)	1(0)	1(0)	
	1–2½ years	29(74.4)	10(25.6)	0.094(0.019–0.455)	0.303(0.048–1.902)	0.203
	2½-5years	94(83.2)	19(16.8)	0.16(0.036–0.709)	0.367(0.057–2.38)	0.293
	> 5years	36(66.7)	18(33.3	0.065(0.014–0.294)	0.074(0.007–0.78)	0.031*
Missed follow up	Never	136(93.2)	10(6.8)	1 (0)	1(0)	
	Missed 1 times	37(78.7)	10(21.3)	0.272(0.105–0.703)	0.301(0.10–0.90)	0.032*
	Missed 2 times	28(68.3)	13(31.7)	0.158(0.062–0.397)	0.176(0.06–0.513)	0.001*
	Missed above 2 times	20(55.6)	16(44.4)	0.092(0.037–0.23)	0.088(0.028–0.278)	< 0.0001**
Reason for adherence	Drug unavailability	58(96.7)	2(3.3)	1(0)	1(0)	
	Drug adverse effect	20(95.2)	1(4.8)	0.69(0.06-8.0)	0.87(0.064–11.74)	0.917
	Financial shortage	13(76.5)	4(23.5)	0.112(0.019–0.679)	0.175(0.024-1.28)	0.086
	Forgetfulness	123(55.7)	41 [12]	0.103(0.024–0.442)	0.104(0.021–0.523)	0.006*
	Other reason	7(87.5)	1(12.5)	0.241(0.019–3.02)	0.85(0.058–12.36)	0.905

AOR: Adjusted Odds ratio; COR: Crude Odds ratio; CI: Confidence interval; N-number of patients; P-Probability; SPHMMC- Saint Paul`s Hospital Millennium Medical College

* Statistically significant

## Discussion

According to the SMAQ scale, the prevalence of medication adherence among the kidney transplant population was found to be 81.9%. This proportion is high as compared to a previous study done on chronic medication adherence at Tikur Anbessa Hospital [1]. However, this could vary between different medication adherence level measurement startegies like Brief Medication Questionnaire (BMQ), Medical Outcomes Study (MOS), Medication Adherence Scale (MAS), Morisky Adherence Questionnaire eight item (MAQ) [17–21]. And despite all the shortages in the country for kidney transplant service, immunosuppressant medication, investigation modality, and limited skilled professionals, adherence at St. Paul’s hospital was higher than the WHO stated adherence level and other studies [1, 7, 11, 12]. This could be explained by the continuous health education given to patients by the health care providers. In addition, the scarcity of resources for transplant services, forces patients to be adherent, and it is a matter of life and death because the alternative is transplant rejection, which is unacceptable option for most patients due to many limitations.

Duration after transplant above five years, missed follow-up visits, and forgetfulness. All the other factors didn’t show significance in the multivariable analysis. Missing follow up had a strong association with decreased medication adherence levels among kidney transplanted patients as compared to those who never missed follow up visits. Those who missed more than two times were higher (91.2%) less adherent as compared to those who missed two times (82.4%) and one time (69.9%) when they were compared with those who never missed follow up. This could be explained by the fact that if patients have missed their follow-up, it is more likely that they will finish their medication without refilling and hence, will not fully adhere to their medication. This is also supported by other studies conducted in the US [5, 25, 26].

Similarly, the duration after transplant was the other significant associated factor for adherence level; patients with duration after transplant above five years had (92.6%) less adherent as compared to those with duration after transplant one year and below. This can be explained by the fact that, as the long duration after the transplant was increasing so is the treatment intake years. The other important factor that determines medication adherence is forgetfulness. The odds of being adherent in patients responded forgetfulness is lower than the other reasons provided by the patients, like drug unavailability, financial shortage and drug adverse effects. That means, being forgetfulness was associated with 90.6% decreased medication adherence level as compared to the other reasons provided. This is supported by other studies done in the US, Iraq, WHO and Ethiopia [1, 6–8, 11, 22, 27]. There were other factors significantly associated with medication adherence in other studies, like knowledge about medication, financial shortages, perceived health, and others [12–19, 24]. But none of these factors were significantly associated with adherence level in this study.

Medication unavailability is a perceived reason for medication non-adherence for patients with KT because almost all medications are imported and expensive, and hard to find in government hospitals [4, 11]. 

The strength of this study is that it has included most of the represented population which can be used for generalization. The limitation of this study was that patients could have a recall bias due to the long duration of after transplant years, which may reveal a lower prevalence rate than the measured one. And also, the measurements have not been validated, and reliability tests were not done for this particular setup.

## Conclusion

In Ethiopia, the prevalence of medication adherence among kidney transplant patients was optimal. The factors that decreased adherence were missing follow-up, over five years of duration after transplant, and forgetfulness. This suggests that in post kidney transplant patients in St. Paul’s hospital, Ethiopia, despite all the limitations that the patients face, the proportion of medication adherence was higher as compared to other international and domestic studies. The associated factors determined by the study were coherent with the factors identified in other studies. The reason for increased adherence could be attributed to the health education program that the institution has for patients. The health education programs are prior to the surgery, during hospital stay ( peri-operative period), and post transplant period during follow-up period. The content of the education regarding medication adherence is a detailed explanation of the medication name, dosing, frequency of ingestion and adverse effects of the drugs and effects of non-adherence. If patients miss follow-up, a phone call reminder might also be taken as an action.

### Recommendations

The recommendation is to the researchers, to have another study to assess the validity and reliability of the two measurements (SMAQ and BAASIS) with repetitions of the data collection by different health care workers for intra-observer and inter-observer interview variation. And also to do a cause and effect relationship by using a stronger study design based on this study finding.

And for health care providers, they should strictly follow medication adherence among patients. They should strengthen the health education that is being given; and persuade their patients to set a reminder or alarm on their mobile and note pad about their medication intake time and follow-up visits. The other recommendation for HCP is to count pills during follow-up time to trace missed doses, underdoses or overdoses. Patients with more than five years post-transplant should be given special attention to the above recommendations.

For the institution, it should continuously and readily avail of the medication and the investigation modalities, like tacrolimus level. It should encourage and strengthen the service that is being given. It should build a system for regular messaging, tracking patients when they miss follow-up and contacting them through their phone number.

## Data Availability

Data sets used or analyzed during the current study are available without restriction at the request of the corresponding author.

## References

[1] Kefale B, Tadesse Y, Alebachew M, Engidawork E. Management practice, and adherence and its contributing factors among patients with chronic kidney disease at Tikur Anbessa Specialized Hospital: a hospital-based cross-sectional study. PLoS ONE. 2018;13(7):e0200415. 10.1371/journal.pone.0200415. PMID: 30044830; PMCID: PMC6059431. 30044830 10.1371/journal.pone.0200415 PMC6059431

[2] Low JK, Williams A, Manias E, Crawford K. Interventions to improve medication adherence in adult kidney transplant recipients: a systematic review. Nephrol Dial Transpl. 2015;30(5):752–61. 10.1093/ndt/gfu204. Epub 2014 Jun 20. PMID: 24950938. 10.1093/ndt/gfu204 24950938

[3] Okafor UH. Kidney transplant in Nigeria: a single centre experience. Pan Afr Med J. 2016;25:npag. 10.11604/pamj.2016.25.112.7930 PMC5325483 28292075

[4] Seifemichael G, Mahteme B, Engida A, Tekleberhan B, Mekdim T, Kenth W, Leja H, Berhanu W, Hamelemal G, Berahne R, Balkachew N, Wondemagegn G, Zerihun A, Senait F, Mersema A, Fasika T, Aklilu AA, Jeffery DP, Alan L, Momina A. Ethiop Med J. 2020; Suppl 1.

[5] Taber DJ, Fleming JN, Fominaya CE, Gebregziabher M, Hunt KJ, Srinivas TR, Baliga PK, McGillicuddy JW, Egede LE. The Impact of Health Care Appointment Non-Adherence on Graft Outcomes in Kidney Transplantation. Am J Nephrol. 2017;45(1):91–98. 10.1159/000453554. Epub 2016 Dec 2. PMID: 27907919; PMCID: PMC5214896. 10.1159/000453554 PMC5214896 27907919

[6] Patzer RE, Serper M, Reese PP, Przytula K, Koval R, Ladner DP, Levitsky JM, Abecassis MM, Wolf MS. Medication understanding, non-adherence, and clinical outcomes among adult kidney transplant recipients. Clin Transplant. 2016 Oct;30(10):1294–1305. 10.1111/ctr.12821. Epub 2016 Aug 29. PMID: 27447351; PMCID: PMC5061615. 10.1111/ctr.12821 PMC5061615 27447351

[7] Burkhart PV, Sabaté E. Adherence to long-term therapies: evidence for action. J Nurs Scholarsh. 2003;35(3):207. PMID: 14562485. 14562485

[8] Nevins TE, Nickerson PW, Dew MA. Understanding medication nonadherence after kidney transplant. J Am Soc Nephrol. 2017;28(8):2290–301. 10.1681./ASN.2017020216. Epub 2017 Jun 19. PMID: 28630231; PMCID: PMC5533244. 28630231 10.1681/ASN.2017020216 PMC5533244

[9] Gordon EJ, Ladner DP, Caicedo JC, Franklin J. Disparities in kidney transplant outcomes: a review. Semin Nephrol. 2010;30(1):81–9. 10.1016/j.semnephrol.2009.10.009. PMID: 20116652; PMCID: PMC2818243. 20116652 10.1016/j.semnephrol.2009.10.009 PMC2818243

[10] Muller E. Transplantation in Africa - an overview. Clin Nephrol. 2016 Supplement 1;86 (2016)(13):90–95. 10.5414/CNP86S125. PMID: 27879190. 10.5414/CNP86S125 27879190

[11] Ejigu AM, Ahmed MM, Mengistu YT. Nephrology in Ethiopia. In: Moura-Neto JA, Divino-Filho JC, Ronco C, editors. Nephrology Worldwide. Cham: Springer; 2021. Available from: 10.1007/978-3-030-56890-0_4.

[12] Shih MH, Tsai CH. [Factors associated with adherence to immunosuppressive therapyamong transplant recipients]. Hu Li Za Zhi. 2014 Aug;61(4):21–5. Chinese. 10.6224/JN.61.4.21. PMID: 25116311. 10.6224/JN.61.4.21 25116311

[13] Prihodova L, Nagyova I, Rosenberger J, Majernikova M, Roland R, Groothoff JW, van Dijk JP. Adherence in patients in the first year after kidney transplantation and its impact on graft loss and mortality: a cross-sectional and prospective study. J Adv Nurs. 2014 Dec;70(12):2871–83. 10.1111/jan.12447. Epub 2014 May 22. PMID: 24853863. 10.1111/jan.12447 24853863

[14] Cukor D. Adherence in Kidney Transplant Recipients. Am J Nephrol. 2017;45(1):89–90. 10.1159/000453555. Epub 2016 Dec 2. PMID: 27907912. 10.1159/000453555 27907912

[15] Peterson KJ, Serrano O, Odegard M, Mongin SJ, Berglund D, Vock DM et al. Outcomes for Somali immigrant kidney transplant recipients in a large-volume transplant center. Transplantation Reports. 2020 Dec;5(4):100066. 10.1016/j.tpr.2020.10006

[16] Wu DA, Robb ML, Forsythe JLR, Bradley C, Cairns J, Draper H, Dudley C, Johnson RJ, Metcalfe W, Ravanan R, Roderick P, Tomson CRV, Watson CJE, Bradley JA, Oniscu GC. Recipient Comorbidity and Survival Outcomes After Kidney Transplantation: A UK-wide Prospective Cohort Study. Transplantation. 2020 Jun;104(6):1246–1255. 10.1097/TP.0000000000002931. PMID: 31449188. 10.1097/TP.0000000000002931 31449188

[17] Costa E, Giardini A, Savin M, Menditto E, Lehane E, Laosa O, Pecorelli S, Monaco A, Marengoni A. Interventional tools to improve medication adherence: review of literature. Patient Prefer Adherence. 2015 Sep 14;9:1303–14. 10.2147/PPA.S87551. PMID: 26396502; PMCID: PMC4576894. 10.2147/PPA.S87551 PMC4576894 26396502

[18] Kleinsinger F. The Unmet Challenge of Medication Nonadherence. Perm J. 2018;22:18–033. 10.7812/TPP/18-033. PMID: 30005722; PMCID: PMC6045499. 10.7812/TPP/18-033 PMC6045499 30005722

[19] Lam WY, Fresco P. Medication Adherence Measures: An Overview. Biomed Res Int. 2015;2015:217047. 10.1155/2015/217047. Epub 2015 Oct 11. PMID: 26539470; PMCID: PMC4619779. 10.1155/2015/217047 PMC4619779 26539470

[20] Sakthong P, Chabunthom R, Charoenvisuthiwongs R. Psychometric properties of the Thai version of the 8-item Morisky Medication Adherence Scale in patients with type 2 diabetes. Ann Pharmacother. 2009 May;43(5):950–7. 10.1345/aph.1L453. Epub 2009 Apr 14. PMID: 19366872 10.1345/aph.1L453 19366872

[21] Agala CB, Fried BJ, Thomas JC, Reynolds HW, Lich KH, Whetten K, Zimmer C, Morrissey JP. Reliability, validity and measurement invariance of the Simplified Medication Adherence Questionnaire (SMAQ) among HIV-positive women in Ethiopia: a quasi-experimental study. BMC Public Health. 2020 Apr 28;20(1):567. 10.1186/s12889-020-08585-w. PMID: 32345253; PMCID: PMC7189687 10.1186/s12889-020-08585-w PMC7189687 32345253

[22] de Oliveira-Filho AD, Morisky DE, Neves SJ, Costa FA, de Lyra DP Jr. The 8-item Morisky Medication Adherence Scale: validation of a Brazilian-Portuguese version in hypertensive adults. Res Social Adm Pharm. 2014 May-Jun;10(3):554–61. 10.1016/j.sapharm.2013.10.006. Epub 2013 Oct 26. PMID: 24268603. 10.1016/j.sapharm.2013.10.006 24268603

[23] Ortega Suárez FJ, Sánchez Plumed J, Pérez Valentín MA, Pereira Palomo P, Muñoz Cepeda MA, Lorenzo Aguiar D, Grupo de Estudio Vatren. Validation on the simplified medication adherence questionnaire (SMAQ) in renal transplant patients on tacrolimus. Nefrologia. 2011;31(6):690–6. English, Spanish. 10.3265/Nefrologia.pre2011.Aug.10973. PMID: 22130285. 10.3265/Nefrologia.pre2011.Aug.10973 22130285

[24] Demian MN, Shapiro RJ, Thornton WL. An observational study of health literacy and medication adherence in adult kidney transplant recipients. Clin Kidney J. 2016 Dec;9(6):858–865. 10.1093/ckj/sfw076. Epub 2016 Aug 31. PMID: 27994867; PMCID: PMC5162408 10.1093/ckj/sfw076 PMC5162408 27994867

[25] Low JK, Manias E, Crawford K, Walker R, Mulley WR, Toussaint ND, Dooley M, Kennedy E, Smith CL, Nalder M, Yip D, Williams A. Improving medication adherence in adult kidney transplantation (IMAKT): A pilot randomised controlled trial. Sci Rep. 2019 May 22;9(1):7734. 10.1038/s41598-019-44002-y. PMID: 31118485; PMCID: PMC6531445. 10.1038/s41598-019-44002-y PMC6531445 31118485

[26] Marsicano Ede O, Fernandes Nda S, Colugnati F, Grincenkov FR, Fernandes NM, De Geest S, Sanders-Pinheiro H. Transcultural adaptation and initial validation of Brazilian-Portuguese version of the Basel assessment of adherence to immunosuppressive medications scale (BAASIS) in kidney transplants. BMC Nephrol. 2013 May 21;14:108. 10.1186/1471-2369-14-108. PMID: 23692889; PMCID: PMC3665586 10.1186/1471-2369-14-108 PMC3665586 23692889

[27] Jones D, Cook R, Rodriguez A, Waldrop-Valverde D. Personal HIV knowledge, appointment adherence and HIV outcomes. AIDS Behav. 2013 Jan;17(1):242–9. 10.1007/s10461-012-0367-y. PMID: 23143751; PMCID: PMC3549052 10.1007/s10461-012-0367-y PMC3549052 23143751

[28] Denhaerynck K, Dobbels F, Košťálová B, De Geest S; BAASIS consortium. Psychomotor Properties of the BAASIS: A Meta-analysis of individual Participant Data [ published online ahead of print, 2023 March].

[29] Haupenthal F, Doberer K, Bauernfeind F, Ebenberger K, Kainz A, De Geest S, Bond G. Predictive validity of the Basel Assessment of Adherence to immunosuppressive Medication Scale (BAASIS©) after renal transplantation: May 2024.

[30] https://baasis.nursing.unibas.ch/

